# Triple planetary crisis: why healthcare professionals should care

**DOI:** 10.3389/fmed.2024.1465662

**Published:** 2024-09-18

**Authors:** Fathima Rizka Ihsan, Jacqueline G. Bloomfield, Lynn V. Monrouxe

**Affiliations:** ^1^Faculty of Medicine and Health, Sydney School of Health Sciences, The University of Sydney, Camperdown, NSW, Australia; ^2^Faculty of Medicine and Health, Sydney Nursing School, The University of Sydney, Camperdown, NSW, Australia

**Keywords:** planetary health, climate change, pollution, biodiversity, healthcare, eco-anxiety, ecological crisis

## Abstract

Humanity currently faces an ecological crisis with devastating consequences to all living species. While climate change is estimated to lead to 250,000 extra deaths per year between 2030 and 2050, pollution is known to cause 9 million premature deaths: a figure much greater than the deaths caused by AIDS, tuberculosis and malaria combined. The healthcare sector is both burdened by, and contributes to, the impact of climate change and environmental degradation. Amidst glaring evidence of the interdependence of human health and the eco system, there is an urgent call for healthcare professionals to concern themselves with the triple planetary threat humanity currently faces. Without immediate mitigative measures, the future seems uncertain. Some healthcare systems at local, national and global levels have taken numerous initiatives to address, mitigate and adapt to these changes, however, these are not sufficient. A lack of awareness among healthcare professionals of the ecological crisis, its interconnectedness, and the role of healthcare in it, plays a significant role in the lack responsibility of healthcare professionals in this space. Therefore, this paper presents a discussion of the current landscape of the triple threat of climate change, loss of biodiversity, and pollution, while emphasising the contribution of healthcare professionals to it. Furthermore, interrelated concepts such as planetary health and eco-anxiety are briefly discussed. This perspective paper also presents several key prospective research areas that may lay the foundation for motivating healthcare professionals to play an active role in preventing and mitigating the ecological crises humanity currently faces.

## Introduction

The “triple planetary crisis” refers to three interconnected and critical environmental issues facing our planet: climate change, biodiversity loss, and pollution (1). Climate change is having monumental effects on a global scale. Its detrimental impact on human health and ecosystems is escalating, so much so that climate change has been recognized as the leading threat to human health in the 21st century (2, 3). The impact of pollution on human health has exceeded that of several diseases combined (4) and the present rate of decline in biodiversity is poorly known (5). The environmental and human health effects of the ecological crises have only begun to unravel, and the forecast seems devastating. The emotional implications are taking a toll on the mental well-being of the population (6, 7). While the global healthcare sector has a central role of improving population health, at the same time it emits greenhouse gases equivalent to 514 coal-fired power plants (8): leading to a situation whereby the healthcare sector both contributes to, and is burdened by, the ongoing environmental degradation. Thus reinforcing a vicious cycle. Despite this immense negative role, the healthcare sector lags in its action to mitigate its impact on the environment. While some healthcare professionals are of the view that this is beyond their responsibilities (9), a lack of awareness of the triple planetary crisis and the role of healthcare in it is quite apparent among healthcare professionals (10–13). This paper aims to bring together these different yet interconnected constructs and make a case for the healthcare workforce to take on an active role in this daunting fight against the triple planetary crisis.

## Ecological crisis

### Climate change

Climate change is having an escalating detrimental impact on human health and ecosystems (14). Climate change has been referred to as persistent (over decades) “*changes in the mean and/or the* var*iability of its properties*” (15). However, issues around causality of climate change have been variously attributed. On the one hand it has been argued that climate change “*may be due to natural internal processes or external forcings such as modulations of the solar cycles, volcanic eruptions and persistent anthropogenic changes in the composition of the atmosphere or in land use*” (15). Here, causality is deemed neutral or even in equal measures between natural and human factors. On the other hand, the construct of climate change has been exclusively used to refer to direct or indirect “*human activity that alters the composition of the global atmosphere **and which is in addition to** natural climate* var*iability observed over comparable time periods*” (our emphasis) [(16), p. 3]. From this perspective, climate change is referred to as changes caused by human-led activities only. Indeed, it has been argued that human-led activities have led to climate change much more excessively than natural processes (17).

The key change that sets off the chain of catastrophic events of climate change is the rise in global surface temperature. Human-induced global warming is presently increasing at a rate of 0.2°C per decade (18). With an average temperature of 1.5°C above pre-industrial era, 2023 recorded the warmest year since direct observations began (19). A rise in the temperature from 1.5°C to 2°C is forecast to have serious negative impacts on all life on earth. This ranges from an increase in water scarcity by 14%, a twofold rise in the population exposed to extreme heat, a tenfold increase in arctic melting, the near disappearance of coral reefs, a 50% drop in crop output, diminished seafood yield, and a 50% decrease in our loss of biodiversity including vertebrates, plants and insects (20). The release of greenhouse gases (GHGs) is the main way in which humans contribute to climate change as GHGs capture heat resulting in a rise in global temperature that leads to a cascade of events. Furthermore, the time lag between a pulse of GHGs and its warming effects is about a decade (21). This suggests that the global community is yet to experience the true effects of our current actions.

### Climate change and the healthcare sector

GHG emission sources are mostly electricity and heat, transportation, and agricultural processes that release carbon dioxide, methane and nitrous oxide and deforestation that lead to a loss of major carbon sinks (22). It is said that if the global health sector were a country, it would be the fifth largest carbon footprint generator in the world (23). In 2016, it was estimated that 4.6% of all greenhouse gas emissions worldwide originated from the healthcare sector while this figure was higher in Australia (7%) and the United States (9.8%) (24). Of the total greenhouse gas emissions from the healthcare sector, 71% were from its supply chain (24).

It is evident that climate change is causing environmental catastrophes on a global scale and significantly impacting all living beings. It is also affecting social and environmental determinants of health that lead to, among many others, an increase in the prevalence and severity of respiratory diseases including: asthma, vector-borne diseases, heat-wave-related deaths, food and water-borne diseases and mental health problems (25–27). Furthermore, adverse events directly impact access to healthcare and increases mortality (25). The World Health Organization (WHO) estimates that there will be an additional 250,000 deaths per year due to the implications of climate change between 2030 and 2050 (28). While it is recognized as a crisis multiplier due to its potential to aggravate existing threats, climate change has been declared the leading threat to human health in the 21st century by the WHO and The Lancet (2, 3). This positions the health sector in a complexity of being both burdened by, and contributing to, the impact of climate change and environmental degradation.

### Pollution

Pollution in the forms of water, soil and air has greatly risen in recent times and is causing disastrous consequences for human health and the environment (4, 29). The Lancet Commission on Pollution and Health reported that all forms of pollution combined were responsible for an estimated 9 million premature deaths in 2015 which is three times greater than deaths caused by AIDS, Tuberculosis and Malaria combined (30). Water pollution caused by human activities and natural factors such as urbanization, inappropriate industrial waste disposal, climate change, poor water supply and sewage treatment and agriculture activities is responsible for an estimated 829,000 deaths per year (31, 32).

### Pollution and the healthcare sector

Concerningly, air pollution is responsible for 6.4 million deaths (30) and air pollution is on track to increase by two-fold by 2050 (33). Diverse components of the healthcare system are contributing to pollution ranging from the cafeteria to the operating theatre. Major toxic air pollutants are released from healthcare facilities including anesthetic gases, boilers, cooling, ventilation and incineration of medical waste and from the supply chain of healthcare comprising services and goods such as the pharmaceutical organizations (34–36). While soil pollution by heavy metals, toxic organic waste and nano and micro-plastics, impacts the agri-food system, it also affects soils’ ability to store water and remove water contaminants (37). It has been recorded that an estimate of 87,000 tonnes of PPE and 144,000 tonnes of additional waste such as syringes and needles were generated during the first 1.5 years of the Covid-19 pandemic (38). Pollution of this vital constituent of the planet’s infrastructure disrupts many ecosystem services and all forms of life on earth (37).

Untreated medical waste disposed of in landfills leads to contamination of water if improperly designed. Furthermore, consequences of the comparatively silent threat; chemical pollution, have not yet been adequately quantified, however, its effects of neurotoxicity, reproductive toxicity and immunotoxicity are of great concern (23). Inappropriate handling, storing or disposing of chemical disinfectants of healthcare wastes contributes to this alarming threat. Amidst these distressing statistics there exists a significant disparity between pollutions’ impact on human health and the international resources directed towards its control; less than 2 billion dollars have been dedicated to pollution mitigation which is responsible for an estimate of 9 million deaths whereas more than 25 billion dollars have been designated to tackle AIDS, Malaria and Tuberculosis which lead to an estimate of less than 3 million deaths in total (4). This demonstrates the degree to which the international community views pollution as a serious threat.

### Biodiversity

The Convention on Biological Diversity in 1992 defined biodiversity as “*the* var*iability among living organisms from all sources including, inter alia, terrestrial, marine and other aquatic ecosystems and ecological complexes of which they are part; this includes diversity within species, between species and of ecosystems*” (39). In simpler terms it refers to the variety of life in genes, species, and habitats (40). Biodiversity is known to play a critical role in water purification, maintenance of soil quality and pollination directly affecting food and water essential for human life (41). The protective function of biodiversity has only recently begun to be appreciated. At present the world faces a global decline in biodiversity at a rate that is difficult to quantify and is inadequately recorded (42). While this is also caused by natural factors, the rate at which it occurs due to human activities far exceeds this natural process (42, 43). The main drivers of this include over-exploitation, climate change, invasive species and change in land use mostly due to agricultural expansion to meet the demands of global supply chains (44, 45).

### Biodiversity and the healthcare sector

Biodiversity plays an essential role in drug discovery, biotechnology breakthrough and maintenance of ecosystems: a disruption of which would lead to the spread of diseases (41, 46). In addition to the complex healthcare supply chain, massive energy consumption by healthcare facilities for ventilation, heating and air conditioning purposes, the use of fresh water and waste generation resulting in loss of biodiversity and a significant strain on natural resources (47). Furthermore, evidence suggests that the human immune system is intricately linked to biodiversity exposure with reduced biodiversity being associated to increased prevalence of allergies and chronic inflammatory diseases in the urban population (48). The interdependence of human health and the eco system, amidst such evidence, is undeniable.

## Planetary health and the education of health professions

Environmental activists, scientists, and educators are urging both the public and professionals to take immediate mitigative measures to combat what is now a current problem that threatens the future of civilization (49–51). Many position papers have called for the recognition of the ethical obligation of healthcare professionals to concern themselves with the triple planetary threat humanity faces (52–54) and the inclusion of planetary health in the health professions curricula (55, 56). Planetary health, defined as “*the achievement of the highest attainable standard of health, well-being, and equity worldwide through judicious attention to the human systems-political, economic, and social-that shape the future of humanity and the earth’s natural systems that define the safe environmental limits within which humanity can flourish*” [(57), p. 1978], unifies and acknowledges interdependent relationships between living organisms and their ecosystems.

Successful mitigation of the challenges humanity faces requires trans and interdisciplinary and innovative strategies. Founded on five domains, of which “*the interconnection within nature*” plays a central role [(58), p. 253], planetary health focuses on human health and the natural systems within which humans exist (57). While it primarily focuses on human health, a shift from concepts such as one health and eco health (59), it is centered on the dependency of human health and well-being on planets health. Furthermore, it can be viewed as an “umbrella” field which encompasses existing concepts such as Public, Global and Environmental Health (60). Maintaining planetary health requires an understanding of interrelated natural systems of the earth and the health benefits that arise from conserving and restoring these systems. Therefore, a comprehensive and holistic approach to health, such as planetary health is better suited to address the unprecedented challenges humanity faces at present (48) and this can be in cooperated into health professions curricular by highlighting the need for healthy eco-systems for human health (60). Furthermore, the 17 United Nations Sustainable Development Goals (SGD) address crucial aspects of the environment ranging from climate action to inequalities. As the principles of planetary health and sustainable development goals (SGDs) are well aligned, planetary health may also be viewed as the visualisation of SGDs (61, 62).

However, the current healthcare landscape is rather different. While there are numerous sustainable healthcare initiatives at local, national and global levels, these are trivial considering the extent of the problem (63). Concepts of climate change have been minimally included in health professions curricular globally (64) and, likewise, the principles of planetary health in clinical practice have been applied narrowly (48). Evidence suggests that clinicians and nurses are of the perception that climate action is “*peripheral to their role of a healthcare professional*” [(9), p. 5] and that there are certain professionals whose roles such as public health professionals are better suited than theirs to carry out this work (9). Additionally, some healthcare professionals feel there is minimal support from colleagues and superiors to communicate about climate change to the public and implement mitigative measures (65, 66).

Over the decades, healthcare professionals have played an active role in advocating and leading by example for health concerns such as sanitation and hygiene, tobacco control and the prevention of war (67). Why do healthcare professionals feel different in this instance, especially when the healthcare sector itself plays a key negative role? We argue that the problem is most likely due to a lack of understanding around this complex issue, alongside ignorance of what action is possible for healthcare professionals to take. Subsequently, we believe it is imperative to identify factors that affect healthcare professionals’ sustainability practices. As an individual’s perception, attitude and practices are influenced by contextual and cultural factors (68, 69), for transferability and practical implications, examination of influencing factors across diverse contexts will be valuable.

## Eco-anxiety

With the rapidly growing concern about the ecological crisis, emotions related to it have gained substantial global attention (70). Studies exploring emotions related to the ecological crisis have been conducted by scholars from diverse disciplines in a range of cohorts such as specific communities, climate activists, adolescents, children, climate researchers, environmental educators and students (70–72). Eco-anxiety, a term coined to describe “*anxiety in relation to the ecological crisis*” [(70), p. 2], and climate anxiety, defined as “*forms of anxiety that are considerably related to the climate crisis*” [(70), p. 2], are commonly used interchangeably as labels for examining strong negative emotions related to our ecological crisis in literature (70).

While not implying mental illness, the vulnerability of children and adolescents to this has been reported (71–74). Indeed, a study conducted among adolescent psychiatrists in the UK found that 57% of their participants (comprising Child and Adolescent Psychiatrists in England) had seen a patient who was distressed about ecological issues during the past year (7), while another Australian-based study found that four out of five students felt “*somewhat or very anxious about climate change*” [(6), p. 1]. Furthermore, an American poll of among 4,400 females aged between 18 and 44 years found that 14.3% cited climate change as a major reason (and 20.7% cited it as a minor reason) for not having children (75). Against this background we might legitimately ask whether healthcare professionals are adequately informed and prepared to meet this demand for patient support.

Similarly, it is crucial for healthcare professionals to deal with their own emotions related to the ecological crisis prior to providing therapeutic interventions to their patients. Amidst the clear need for professionals trained in managing patients with eco-anxiety, the literature suggests that there is minimal guidance and scholarly work addressing this aspect (6). There is scarce evidence on the prevalence of eco-anxiety amongst the population of healthcare professions. While a recent study among Italian doctors is a great start (76), work in this space is still in infancy. Accurate assessment of eco-anxiety requires the use of validated tools designed based on the domains of eco-anxiety, therefore we encourage people to engage with the wider literature outside healthcare professions education to develop their thinking in this area.

## Conclusion

With the rising disease burden, shifting population needs and ever evolving technology and treatment modalities, healthcare professionals are overwhelmed with the delivery of quality patient care (77, 78). Existing staff shortages and other resource constraints exasperate carrying out their responsibilities (77, 79, 80). In this context, healthcare professionals may view the ecological crisis beyond their scope of responsibilities and the gradual manifestations of its effects, adding to it. However, we believe that this perspective is detrimental to health in the long run. It is evident that the healthcare sector significantly contributes to the triple planetary crisis which has detrimental consequences on human health. This increases the need for healthcare, as well as the burden on the healthcare sector, which in turn further increases its environmental impact leading to a vicious cycle. With improving population health as its goal, we argue that it is only ethical that the healthcare workforce plays a pivotal role in the fight against these threats to human health.

Building a climate-conscious, resilient, and environmentally sustainable health workforce is imperative. The lack of awareness among the healthcare community regarding the interrelated aspects discussed in this paper and the negative contribution of the healthcare sector to it, seems to play a significant role in the lack of responsibility of healthcare professionals in this space (11–13, 81). Therefore, to shape the attitude of the current and future healthcare workforce to support and implement sustainable healthcare practices, the importance of education cannot be sufficiently emphasized. Globally, only a few educational institutions have incorporated the principles of planetary health into health professions curricula (82). Little is known about the perspectives of health professions’ educators’, who play pivotal and decisive roles in shaping the course of health professions education (83). Thus, understanding their viewpoints toward the principles of planetary health, sustainability, and the integration of these concepts into health professions curricular would lay the foundation in the healthcare sectors’ role in preventing and mitigating our ecological crises. Without this crucial educational shift, the healthcare sector will remain ill-prepared to combat and mitigate the pressing ecological crises of our time.

## Data Availability

The original contributions presented in the study are included in the article/supplementary material, further inquiries can be directed to the corresponding author.
